# Supplementary material for: Does putting down your smartphone make you happier? the effects of restricting digital media on well-being
Source: PLoS One. 2024 Oct 14;19(10):e0306910. doi: 10.1371/journal.pone.0306910 (PMC11472914; doi:10.1371/journal.pone.0306910)
Supplement: S1 File — (DOCX) [file pone.0306910.s001.docx]

Does Putting Down Your Smartphone Make You Happier? The Effects of Restricting Digital Media on Well-Being

# Supplemental Materials

**Table S1**

*Summary of Key Findings in Digital and Social Media Research*

| **Media** | **Author(s) & Year** | **Method** | ***N*** | **Relevant Outcome(s)** | **Key Finding(s)** |  |
| --- | --- | --- | --- | --- | --- | --- |
| Digital | Orben & Przybylski (2019a) | Correlational | 355,358 | Well-being (–) (e.g., happiness, self-esteem) | Small negative effect of digital media use on well-being—accounting for no more than 0.4% of the variance in well-being. Comparable to benign activities like wearing glasses and eating potatoes. |  |
| Digital | Orben & Przybylski (2019b) | Correlational | 17,247 | Well-being (–) (e.g., mood, self-esteem) | Found small negative correlations between digital screen use—both throughout the day and specifically before bedtime—and the well-being of adolescents. |  |
| Digital | Twenge, Joiner, Rogers, & Martin (2018) | Correlational | 506,820 | Depressive symptoms (+), suicide-related outcomes (+), & suicide rates (+) | Adolescent depressive symptoms, suicide-related outcomes, and suicide rates increased after 2010. Adolescents who spent more time on screen activities were more likely to report mental health issues and adolescents who spent more time on nonscreen activities were less likely. |  |
| Digital | Twenge, Martin, & Campbell (2018) | Correlational | 1.1 million | Happiness (–), life satisfaction (+),  & self-esteem (–) | Adolescent well-being decreased after 2012. Adolescents who spent more time on screen activities and less time on nonscreen activities had lower well-being. |  |
| Digital | Walsh, Okabe-Miyamoto, Regan, Twenge, & Lyubomirsky (2021) | Correlational | 414 | Depression (+), life satisfaction (–), loneliness (+), negative affect (+), positive affect (–), & self-esteem (–) | Objective smartphone use and self-reported use (including social media app time) are negatively related to overall well-being. |  |
| Digital | Kushlev, Proulx, & Dunn (2016) | Correlational & Experimental | 221 | Hyperactivity (–), inattention (+), life satisfaction (–), meaning in life (–), & social connectedness (o) | Participants reported higher levels of inattention and hyperactivity when smartphone notifications were turned on, relative to when notifications were turned off. |  |
| Digital | Brailovskaia, Delveaux, John, Wicker, Noveski, Kim, Schillack, & Margraf (2022) | Experimental | 619 | Anxiety symptoms (–), depressive symptoms (–), life satisfaction (+),  & physical activity (+) | Reducing smartphone use decreased depressive and anxiety symptoms and improved life satisfaction and physical activity. Less time spent on one's smartphone led to greater well-being and a healthier lifestyle. | |
| Digital | Cheever, Rosen, Carrier, & Chavez (2014) | Experimental | 158 | Anxiety (+) | Participants felt significantly more anxious over time when their smartphones were taken away. | |
| Digital | Clayton, Leshner, & Almond (2016) | Experimental | 40 | Anxiety (+), blood pressure (+),  & heart rate (+) | When iPhone users could not answer their ringing iPhones during a word search puzzle, they experienced increases in heart rate, blood pressure, and anxiety. | |
| Digital | Cutonio & Nees (2017) | Experimental | 93 | Anxiety (o) | Anxiety did not differ between participants with smartphone access or without it while working on their own, self-chosen class materials for 60 minutes. | |
| Digital | Dwyer, Kushlev, & Dunn (2017) | Experimental | 423 | Affect (–), distraction (+), interest/enjoyment (–), & social connectedness (–) | Relative to those who placed their phones in a closed container at dinner, diners who kept their phones reported more distraction, as well as lower interest, enjoyment, and well-being during dinner | |
| Digital | Eide, Aarestad, Andreassen, Bilder, & Pallesen (2018) | Experimental | 127 | Affect (o), fear of missing out (+),  & withdrawal symptoms (+) | Relative to control, participants assigned to restrict their smartphone use experienced higher levels of withdrawal symptoms and fear of missing out. No significant main effect of condition for positive and negative affect. | |
| Digital | Fitz, Kushlev, Jagannathan, Lewis, Paliwal, & Ariely (2019) | Experimental | 237 | Anxiety (o), depression (o), inattention (–), mindfulness (o), mood (+), negative affect (–), social connectedness (o), & stress (–) | Compared to those in the control condition (default notification settings), participants whose smartphone notifications were batched three times a day felt more attentive, in a better mood, and less stress. | |
| Digital | Kushlev, Proulx, & Dunn (2017) | Experimental | 274 | Mood (+), interest/enjoyment (–),  & social connectedness (–) | Relative to those using their smartphones, students not using them arrived at an unknown building feeling more socially connected, but it took them longer to find the building and the difficulty of the task appeared to make them less happy. | |
| Digital | Ward, Duke, Gneezy, & Bos (2017) | Experimental | 844 | Cognitive capacity (–) | When participants were successful at maintaining their attention, the mere presence of their smartphone reduced available cognitive capacity. Such cognitive costs were worse for those highly dependent on their smartphones. | |
| Social | Allcott, Braghieri, Eichmeyer, & Gentzkow (2019) | Experimental | 1,661 | Subjective well-being index (+): including anxiety (–), depression (–), happiness (+), life satisfaction (+), loneliness (o), & positive affect (o) | Deactivating Facebook significantly increased subjective well-being, with the largest and most significant effects on anxiety, depression, happiness, and life satisfaction. | |
| Social | Hunt, Marx, Lipson, & Young (2018) | Experimental | 143 | Anxiety (o), depression (–), fear of missing out (o), interpersonal support (o), loneliness (–), psychological well-being (o), self-esteem (o) | Limiting use of major social media platforms reduced loneliness and depression over three weeks. Other outcomes (interpersonal support, fear of missing out, anxiety, self-esteem, and psychological well- being) were non-significant. | |
| Social | Przybylski, Wilbert, & Netta (2021) | Experimental | 600 | day satisfaction (–), negative affect (o), positive affect (o), self-esteem (o), & social relatedness need satisfaction (–) | Abstaining from social media did not improve well-being and sometimes harmed feelings of social relatedness and satisfaction with one's day. | |
| Social | Tromholt (2016) | Experimental | 1,095 | Affective well-being/emotions (+)  & life satisfaction (+) | Taking a break from Facebook significantly improved well-being, especially for heavy and passive users. | |
| Social | Verduyn, Lee, Park, Shablack, Orvell, Bayer, Ybarra, Jonides, & Kross (2015) | Experimental | 84 | Affective well-being (–) | Participants assigned to use Facebook passively (rather than actively) experienced declines in affective well-being over time. | |
| Social | Arad, Barzilay, & Perchick (2017) | Quasi-experimental | 144 | Happiness (–) &  social comparison (+) | Workers had their Facebook usage differentially restricted in a quasi-natural experiment. Facebook usage increased social comparison and reduced happiness, especially among younger employees and those who perceived others as having more positive experiences. | |

#

# *Note.* (−) indicates a negative relation/effect between media use and outcome. (+) indicates a negative relation/effect between media use and outcome. (o) indicates a neutral or non-significant relation/effect between media use and outcome.

# Condition Instructions

**Digital Diet Condition Instructions**We all have small habits that can have a big impact on our daily lives and the world around us. This week, we want you to **restrict your iPhone screen time** (such as time spent on gaming, social media, entertainment, and online news apps). You may use your iPhone for necessary daily activities, such as for GPS navigation, work, school, or to call or message friends or family. But we would like you to limit how much time you spend on your iPhone as much as possible. The more you can limit your overall screen time, the better.  We want you to do your best to restrict any non-necessary screen time.

These are the apps that it would be OK to use only as absolutely necessary (at most a few minutes at a time):

- Phone Messaging apps (e.g., Messages, Messenger, WhatsApp)
- Email apps (e.g., Apple Mail, Gmail)
- GPS/Navigation (e.g., Apple Maps, Google Maps, Waze)
- Weather
- Calendar
- Calculator
- Contacts
- Camera
- Notes
- Other apps you need to obtain necessary information or to do necessary school/work/personal tasks

Please do NOT use these non-necessary apps (or use them as little as possible) this week:

- Social media apps (e.g., Facebook, Twitter, Instagram, Snapchat)
- Gaming apps (e.g., Minecraft, Candy Crush, Angry Birds)
- Entertainment apps (e.g., Netflix, Hulu, HBO)
- News apps (e.g., Apple News, CNN,  Buzzfeed)
- Web browsing apps (e.g., Safari, Chrome) [Unless you need to obtain necessary info]
- Dating apps (e.g., Tinder, OkCupid, Match.com)
- Exercise, health, and relaxation apps (e.g., Fitbit, Lose It!, Calm)
- Reading/books apps (e.g., iBooks, Audible, Amazon Kindle)
- Education apps (e.g., Khan Academy, Duolingo)

Restricting your screen time this week can be made easier by doing some of the following:

- Set a Screen Time app limit of 1 min for all apps and add necessary apps (such as Phone, Messages) to "Always Allowed"
- Delete non-necessary apps off your phone
- Turn off push notifications for non-necessary apps
- Place non-necessary apps into a separate folder on your phone and place that folder on a screen you don’t usually look at
- Log out of non-necessary apps on your iPhone

Please limit your iPhone usage/screen time as much as possible this week—starting tomorrow when you wake up and continuing until your next lab visit. These instructions will be emailed to you to make them easier to follow them throughout the week.

**Social Diet Condition Instructions**

We all have small habits that can have a big impact on our daily lives and the world around us. This week, we want you to **restrict your social media use** as much as possible. Specifically, stay off social media apps/sites (such as Facebook, Instagram, Twitter, and Snapchat)  on your iPhone, computer, iPad, and other e-devices this week. 
  
Examples of social media apps/sites/services that we would like you to avoid entirely include:

- Facebook (NOT including Facebook Messenger or WhatsApp)
- Instagram
- Twitter
- Snapchat
- Google+
- Pinterest
- LinkedIn
- YouTube
- Tumblr
- Sina Weibo
- WeChat
- Naver
- Line
- Qzone
- Kakao Talk
- Dating apps (such as OkCupid, Coffee Meets Bagel, Bumble, Tinder, Grindr, Hinge, Match.com, eHarmony, PlentyOfFish/POF Dating, etc.)

Restricting your social media usage this week can be made easier by doing some of the following:

- Set a Screen Time app limit for 1 min for Social Networking apps
- Delete social media apps off your phone
- Turn off push notifications for those apps
- Place social media apps into a separate folder on your phone and place that folder on a screen you don’t usually look at
- Remove social media bookmarks from your computer web browser
- Log out of social media sites on your devices

We request that you do not look at social media at all this week. However, you may log-in to a service such as Facebook briefly if you need to obtain specific information (e.g., check details for an event), but we ask that you then log-out immediately.

Please limit your social media usage as much as possible this week—starting when you wake up tomorrow until your next lab visit. These instructions will be emailed to you to make them easier to follow throughout the week.

**Water Diet Condition Instructions**

We all have small habits that can have a big impact on our daily lives and the world around us. This week, we want you to **restrict your water usage**, such as by taking shorter showers and using less water when you wash dishes or brush your teeth. However, please do not change the amount of water that you *drink*.

We would like you to conserve the water you use as much as possible. Here are some things we recommend that you do this week:

- Turn off the water when you are not using it. Don’t let it run while you brush your teeth, shave, or wash your hands, dishes, or fruit and vegetables.
- Take shorter showers. Try to cut 1 to 5 minutes off your shower time
- Take baths instead of showers. If you like to linger, a partially filled tub uses less water than a shower.
- Use appliances efficiently. Run full loads in the dish or clothes washer, or, if your appliance has one, use a load selector (e.g., “low water”).
- Water the lawn and garden only when necessary. Early morning or evening are the best times.
- Wash your car sensibly. Clean the car with a pail of soapy water and use the hose only for a quick rinse.

Please limit your water usage (but not how much you drink) as much as possible this week — starting when you wake up tomorrow and continuing until your next lab visit. These instructions will be emailed to you to make them easier to follow throughout the week.

**No Diet Condition Instructions**

[Participants did not receive any condition instructions. They just completed measures.]

**Table S2**

*Means and Standard Deviations by Condition at T_1_ and T_2_ (N = 338)*

|  | Digital Diet | Social Diet | Water Diet | No Diet |
| --- | --- | --- | --- | --- |
| Outcome | *M (SD)* | *M (SD)* | *M (SD)* | *M (SD)* |
| T_1_ SR Digital Media Time | 312.62 (131.8) | 340.19 (135.35) | 327.91 (164.77) | 330.54 (169.34) |
| T_2_ SR Digital Media Time | 199.64 (115.98) | 301.3 (162.68) | 352.2 (196.16) | 384.86 (203.56) |
| T_1_ Obj Digital Media Time | 323.25 (100.28) | 330.67 (107.83) | 333.89 (114.55) | 328.21 (108.28) |
| T_2_ Obj Digital Media Time | 211.37 (105.78) | 275.25 (104.92) | 340.75 (117.44) | 315.75 (113.52) |
| T_1_ SR Social Media Time | 228.01 (129.98) | 242.1 (113.97) | 228.04 (109.73) | 237.6 (105.14) |
| T_2_ SR Social Media Time | 112.32 (106.53) | 89.91 (101.2) | 228.18 (136.09) | 247.75 (149.29) |
| T_1_ Obj Social Media Time | 101.28 (58.82) | 114.71 (62.47) | 117.67 (61.61) | 127.68 (70.47) |
| T_2_ Obj Social Media Time | 57.71 (59.09) | 50.32 (50.27) | 120.7 (68.08) | 117.31 (61.77) |
| T_1_ Brief Happiness | 2.03 (0.46) | 1.85 (0.58) | 1.92 (0.5) | 1.98 (0.48) |
| T_2_ Brief Happiness | 2.08 (0.48) | 2.03 (0.6) | 1.93 (0.6) | 2.1 (0.54) |
| T_1_ Brief Satisfaction | 5.28 (1.09) | 4.91 (1.26) | 4.82 (1.35) | 5.15 (1.2) |
| T_2_ Brief Satisfaction | 5.43 (1.07) | 5.25 (1.21) | 5.02 (1.32) | 5.29 (1.3) |
| T_1_ Positive Emotions | 4.59 (0.96) | 4.46 (1.03) | 4.22 (1.17) | 4.5 (1.25) |
| T_2_ Positive Emotions | 4.75 (0.99) | 4.54 (1.14) | 4.45 (1.19) | 4.85 (1.13) |
| T_1_ Negative Emotions | 2.87 (0.87) | 2.94 (0.88) | 3.03 (0.99) | 3.26 (1) |
| T_2_ Negative Emotions | 2.63 (0.89) | 2.95 (1.03) | 2.92 (1.02) | 2.87 (1.11) |
| T_1_ Life Satisfaction | 4.59 (1.23) | 4.52 (1.09) | 4.35 (1.23) | 4.65 (1.08) |
| T_2_ Life Satisfaction | 5.02 (1.08) | 4.8 (1.06) | 4.45 (1.33) | 4.76 (1.16) |
| T_1_ Mindful Attention | 3.78 (0.98) | 3.77 (0.9) | 3.58 (0.9) | 3.42 (0.91) |
| T_2_ Mindful Attention | 4.15 (0.83) | 4.06 (0.91) | 3.62 (0.95) | 3.64 (0.95) |
| T_1_ Autonomy | 3.64 (0.85) | 3.77 (0.71) | 3.54 (0.78) | 3.82 (0.79) |
| T_2_ Autonomy | 3.96 (0.69) | 3.81 (0.75) | 3.63 (0.82) | 3.88 (0.79) |
| T_1_ Competence | 3.39 (0.86) | 3.43 (0.83) | 3.34 (0.76) | 3.44 (0.83) |
| T_2_ Competence | 3.67 (0.7) | 3.55 (0.82) | 3.42 (0.83) | 3.51 (0.86) |
| T_1_ Connectedness | 3.93 (0.88) | 3.96 (0.81) | 3.74 (0.91) | 3.95 (1) |
| T_2_ Connectedness | 4.01 (0.82) | 3.96 (0.9) | 3.8 (1.03) | 4.04 (0.93) |
| T_1_ Depression | 1.88 (0.77) | 1.99 (0.79) | 2.22 (0.86) | 2.02 (0.83) |
| T_2_ Depression | 1.75 (0.75) | 1.95 (0.74) | 2.14 (0.91) | 1.94 (0.85) |
| T_1_ Loneliness | 2.24 (0.75) | 2.28 (0.76) | 2.48 (0.79) | 2.28 (0.72) |
| T_2_ Loneliness | 2.06 (0.65) | 2.2 (0.82) | 2.44 (0.86) | 2.17 (0.74) |
| T_1_ Self-Esteem | 3.71 (0.7) | 3.63 (0.71) | 3.53 (0.8) | 3.65 (0.78) |
| T_2_ Self-Esteem | 3.98 (0.65) | 3.69 (0.83) | 3.56 (0.8) | 3.73 (0.82) |
| T_1_ Stress | 2.64 (0.74) | 2.82 (0.6) | 2.88 (0.69) | 2.8 (0.65) |
| T_2_ Stress | 2.41 (0.6) | 2.65 (0.72) | 2.78 (0.68) | 2.65 (0.68) |
| T_1_ Health | 75.13 (14.1) | 72.06 (16.43) | 73.47 (13.85) | 71.54 (16.92) |
| T_2_ Health | 77.49 (13.54) | 71.43 (16.99) | 72.66 (17.69) | 73.53 (15.65) |

*Note.* Values outside parentheses indicate outcome means, and parenthetical values represent standard deviations. For ease of interpretation, digital media time and social media time variables are presented in raw form (in minutes). SR = self-reported; Obj = objective.

**Table S3**

*Bivariate Correlations*

*
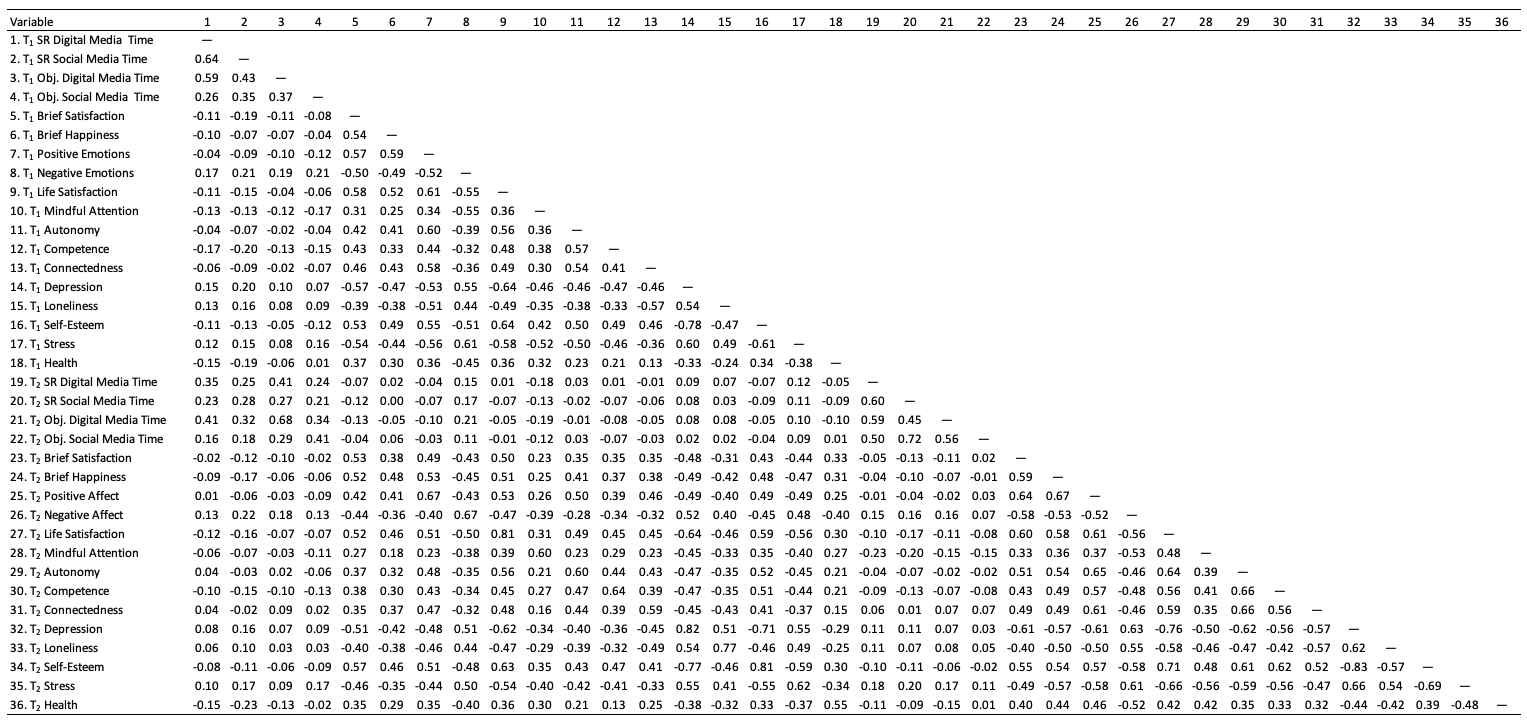
Note.* Correlations (Pearson *r*s) are based on ≤ 338 participants. Correlations .11 and above are significant at *p* < .05. Correlations between .09 and .11 are marginal at p < .1. Digital media time and social media time variables that were right-skewed and kurtotic were log-transformed. SR = self-reported; Obj = objective

**Table S4**

*Manipulation Check Regressed Change Models*

|  |  |  |  | Partial *r* 95% CI | |  |
| --- | --- | --- | --- | --- | --- | --- |
| Manipulation Check Variable  by Comparison | *b* | *b SE* | Partial *r* | *LL* | *UL* | *p* |
| **Self-Reported Digital Media Time** |  |  |  |  |  |  |
| H1. Digital Diet vs. Water Diet | -0.68 | 0.09 | -0.5 | -0.58 | -0.4 | < .001 |
| H1. Digital Diet vs. No Diet | -0.76 | 0.1 | -0.53 | -0.62 | -0.42 | < .001 |
| H1. Digital Diet vs. Both Controls | -0.71 | 0.07 | -0.51 | -0.58 | -0.43 | < .001 |
| H2. Social Diet vs. Water Diet | -0.22 | 0.07 | -0.23 | -0.36 | -0.09 | 0.002 |
| H2. Social Diet vs. No Diet | -0.31 | 0.08 | -0.31 | -0.44 | -0.15 | < .001 |
| H2. Social Diet vs. Both Controls | -0.26 | 0.06 | -0.24 | -0.35 | -0.13 | < .001 |
| E. Digital Diet vs. Social Diet | -0.46 | 0.12 | -0.32 | -0.45 | -0.17 | < .001 |
| **Objective Digital Media Time** |  |  |  |  |  |  |
| H1. Digital Diet vs. Water Diet | -124.67 | 11.53 | -0.63 | -0.69 | -0.55 | < .001 |
| H1. Digital Diet vs. No Diet | -104.64 | 12.73 | -0.56 | -0.64 | -0.46 | < .001 |
| H1. Digital Diet vs. Both Controls | -116.27 | 10.48 | -0.57 | -0.63 | -0.49 | < .001 |
| H2. Social Diet vs. Water Diet | -60.5 | 11.68 | -0.37 | -0.48 | -0.24 | < .001 |
| H2. Social Diet vs. No Diet | -40.49 | 12.8 | -0.26 | -0.4 | -0.1 | 0.002 |
| H2. Social Diet vs. Both Controls | -52.19 | 10.76 | -0.29 | -0.39 | -0.18 | < .001 |
| E. Digital Diet vs. Social Diet | -64.34 | 13.84 | -0.37 | -0.5 | -0.23 | < .001 |
| **Self-Reported Social Media Time** |  |  |  |  |  |  |
| H1. Digital Diet vs. Water Diet | -1.2 | 0.15 | -0.51 | -0.59 | -0.41 | < .001 |
| H1. Digital Diet vs. No Diet | -1.23 | 0.18 | -0.49 | -0.58 | -0.37 | < .001 |
| H1. Digital Diet vs. Both Controls | -1.21 | 0.12 | -0.52 | -0.59 | -0.44 | < .001 |
| H2. Social Diet vs. Water Diet | -1.61 | 0.16 | -0.61 | -0.68 | -0.53 | < .001 |
| H2. Social Diet vs. No Diet | -1.63 | 0.19 | -0.59 | -0.66 | -0.49 | < .001 |
| H2. Social Diet vs. Both Controls | -1.61 | 0.13 | -0.62 | -0.67 | -0.55 | < .001 |
| E. Digital Diet vs. Social Diet | 0.41 | 0.25 | 0.13 | -0.03 | 0.29 | 0.109 |
| **Objective Social Media Time** |  |  |  |  |  |  |
| H1. Digital Diet vs. Water Diet | -1.19 | 0.15 | -0.5 | -0.59 | -0.4 | < .001 |
| H1. Digital Diet vs. No Diet | -1.12 | 0.18 | -0.46 | -0.57 | -0.34 | < .001 |
| H1. Digital Diet vs. Both Controls | -1.17 | 0.12 | -0.51 | -0.58 | -0.43 | < .001 |
| H2. Social Diet vs. Water Diet | -1.59 | 0.17 | -0.6 | -0.67 | -0.51 | < .001 |
| H2. Social Diet vs. No Diet | -1.52 | 0.19 | -0.57 | -0.65 | -0.46 | < .001 |
| H2. Social Diet vs. Both Controls | -1.56 | 0.13 | -0.6 | -0.66 | -0.53 | < .001 |
| E. Digital Diet vs. Social Diet | 0.45 | 0.27 | 0.15 | -0.03 | 0.31 | 0.096 |

*Note.* Hypothesized condition dummy codes predicting T_2_ scores, controlling for T_1_ scores. Digital media time and social media time variables that were right-skewed and kurtotic were log-transformed. H1 = hypothesis 1 comparisons (Digital Diet vs. Controls); H2 = hypothesis 2 comparisons (Social Diet vs. Controls); E = exploratory comparisons (Digital Diet vs. Social Diet); CI = confidence interval; LL = lower limit; UL = upper limit.

# Second-Order Latent Growth Models

In our second-order latent growth models (SOLGMs; see Figure S1), measurement invariance was imposed in the model. Residuals between the same item over time were correlated. We set the variance of the intercept latent variable to 1 and gave it an intercept of 0. The latent variables representing each time point had 0 residual variance as they were fully predicted by the intercept and slope latent variables. We then extracted values of the slope latent variable and predicted those extracted values from condition dummy codes as described above. SOLGM analyses were only conducted for multi-item variables, and thus are not presented for single items (e.g., objective digital media time, brief happiness). Both the regressed change and SOLGM statistical techniques produced highly similar results. See Table S4 for SOLGM fit statistics and Table S5 for SOLGM results.

**Figure S1**

*Example Second-Order Latent Growth Model*


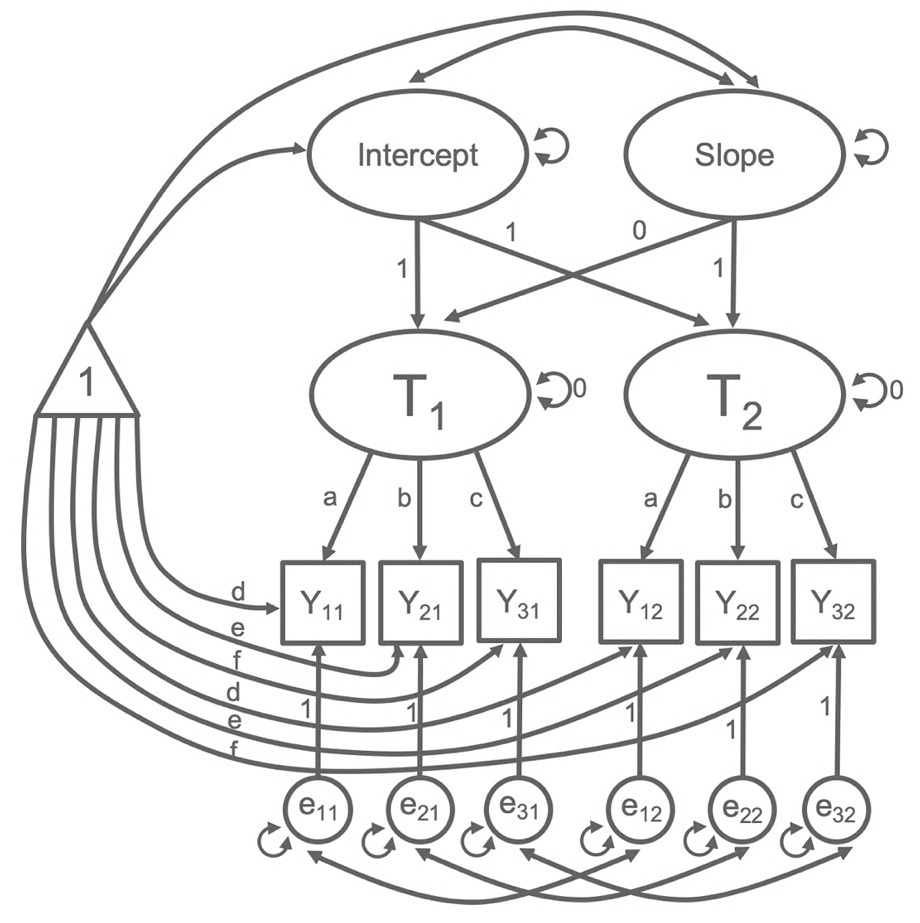


*Note.* Example second-order latent growth model used to model growth in outcome measures (e.g., positive affect, life satisfaction) from T_1_ to T_2_. Factor loadings were constrained to be equal across time. Correlations between the same items over the same duration were constrained to be equal and first-order latent variables had residual variances set to 0.

**Table S5**

*Second-Order Latent Growth Model Fit Statistics*

| Construct | χ^2^ | df | CFI | TLI | RMSEA [90% CI] | SRMR |
| --- | --- | --- | --- | --- | --- | --- |
| **Hypothesis 1. Digital Diet vs. Water Diet:** | | | | | | |
| Positive Emotions | 203.092 | 66 | 0.924 | 0.910 | 0.104 [0.088, 0.121] | 0.073 |
| Negative Emotions | 162.918 | 66 | 0.898 | 0.880 | 0.088 [0.071, 0.105] | 0.066 |
| Life Satisfaction | 71.250 | 44 | 0.982 | 0.977 | 0.057 [0.031, 0.080] | 0.038 |
| Mindful Attention | 85.243 | 66 | 0.950 | 0.937 | 0.070 [0.047, 0.092] | 0.051 |
| Autonomy | 10.466 | 12 | 1.000 | 1.006 | 0.000 [0.000, 0.066] | 0.029 |
| Competence | 25.129 | 12 | 0.968 | 0.944 | 0.076 [0.033, 0.117] | 0.050 |
| Connectedness | 27.107 | 12 | 0.980 | 0.964 | 0.081 [0.040, 0.122] | 0.041 |
| Depression | 148.861 | 66 | 0.951 | 0.942 | 0.081 [0.064, 0.098] | 0.053 |
| Loneliness | 302.270 | 66 | 0.813 | 0.779 | 0.137 [0.121, 0.153] | 0.139 |
| Self-Esteem | 147.226 | 66 | 0.943 | 0.932 | 0.080 [0.063, 0.098] | 0.047 |
| Stress | 42.487 | 26 | 0.970 | 0.959 | 0.058 [0.022, 0.088] | 0.051 |
| Health | 100.324 | 44 | 0.902 | 0.877 | 0.082 [0.061, 0.103] | 0.071 |
| **Hypothesis 1. Digital Diet vs. No Diet:** | | | | | | |
| Positive Emotions | 169.293 | 66 | 0.926 | 0.913 | 0.100 [0.082, 0.119] | 0.058 |
| Negative Emotions | 153.736 | 66 | 0.891 | 0.872 | 0.092 [0.073, 0.111] | 0.066 |
| Life Satisfaction | 92.842 | 44 | 0.953 | 0.942 | 0.084 [0.060, 0.108] | 0.060 |
| Mindful Attention | 88.902 | 66 | 0.932 | 0.915 | 0.081 [0.056, 0.105] | 0.060 |
| Autonomy | 17.079 | 12 | 0.985 | 0.974 | 0.052 [0.000, 0.104] | 0.044 |
| Competence | 17.558 | 12 | 0.984 | 0.972 | 0.054 [0.000, 0.106] | 0.042 |
| Connectedness | 15.746 | 12 | 0.993 | 0.988 | 0.045 [0.000, 0.099] | 0.037 |
| Depression | 120.322 | 66 | 0.957 | 0.949 | 0.073 [0.052, 0.093] | 0.064 |
| Loneliness | 247.956 | 66 | 0.767 | 0.725 | 0.133 [0.116, 0.151] | 0.100 |
| Self-Esteem | 105.791 | 66 | 0.964 | 0.958 | 0.062 [0.039, 0.084] | 0.051 |
| Stress | 43.548 | 26 | 0.953 | 0.934 | 0.066 [0.028, 0.099] | 0.058 |
| Health | 86.263 | 44 | 0.917 | 0.897 | 0.078 [0.054, 0.103] | 0.068 |
| **Hypothesis 1. Digital Diet vs. Both Controls:** | | | | | | |
| Positive Emotions | 235.513 | 66 | 0.936 | 0.924 | 0.097 [0.084, 0.111] | 0.059 |
| Negative Emotions | 179.800 | 66 | 0.919 | 0.905 | 0.080 [0.066, 0.094] | 0.054 |
| Life Satisfaction | 82.220 | 44 | 0.980 | 0.975 | 0.057 [0.037, 0.075] | 0.037 |
| Mindful Attention | 105.095 | 66 | 0.948 | 0.935 | 0.072 [0.054, 0.089] | 0.046 |
| Autonomy | 10.833 | 12 | 1.000 | 1.003 | 0.000 [0.000, 0.057] | 0.026 |
| Competence | 16.979 | 12 | 0.992 | 0.986 | 0.039 [0.000, 0.079] | 0.034 |
| Connectedness | 26.226 | 12 | 0.987 | 0.977 | 0.066 [0.031, 0.101] | 0.032 |
| Depression | 170.993 | 66 | 0.954 | 0.946 | 0.077 [0.063, 0.091] | 0.051 |
| Loneliness | 391.160 | 66 | 0.808 | 0.773 | 0.135 [0.122, 0.148] | 0.133 |
| Self-Esteem | 145.644 | 66 | 0.961 | 0.953 | 0.067 [0.052, 0.081] | 0.041 |
| Stress | 45.773 | 26 | 0.972 | 0.961 | 0.053 [0.026, 0.078] | 0.046 |
| Health | 135.605 | 44 | 0.896 | 0.870 | 0.088 [0.071, 0.105] | 0.069 |
|  |  |  |  |  |  |  |

Table S5 (Continued)

| Construct | χ^2^ | df | CFI | TLI | RMSEA [90% CI] | SRMR |
| --- | --- | --- | --- | --- | --- | --- |
| **Hypothesis 2. Social Diet vs. Water Diet:** | | | | | | |
| Positive Emotions | 180.423 | 66 | 0.934 | 0.922 | 0.098 [0.081, 0.115] | 0.065 |
| Negative Emotions | 135.925 | 66 | 0.928 | 0.915 | 0.076 [0.058, 0.094] | 0.062 |
| Life Satisfaction | 46.848 | 44 | 0.998 | 0.997 | 0.019 [0.000, 0.054] | 0.037 |
| Mindful Attention | 62.521 | 66 | 0.977 | 0.971 | 0.048 [0.014, 0.074] | 0.042 |
| Autonomy | 14.299 | 12 | 0.994 | 0.990 | 0.032 [0.000, 0.086] | 0.041 |
| Competence | 21.360 | 12 | 0.977 | 0.959 | 0.065 [0.009, 0.110] | 0.044 |
| Connectedness | 22.626 | 12 | 0.986 | 0.975 | 0.070 [0.021, 0.113] | 0.035 |
| Depression | 140.265 | 66 | 0.950 | 0.941 | 0.079 [0.061, 0.097] | 0.050 |
| Loneliness | 270.152 | 66 | 0.837 | 0.807 | 0.130 [0.114, 0.147] | 0.147 |
| Self-Esteem | 112.979 | 66 | 0.965 | 0.959 | 0.063 [0.042, 0.082] | 0.046 |
| Stress | 19.360 | 26 | 1.000 | 1.021 | 0.000 [0.000, 0.037] | 0.041 |
| Health | 136.043 | 44 | 0.860 | 0.824 | 0.107 [0.087, 0.128] | 0.089 |
| **Hypothesis 2. Social Diet vs. No Diet:** | | | | | | |
| Positive Emotions | 163.364 | 66 | 0.930 | 0.917 | 0.100 [0.081, 0.120] | 0.056 |
| Negative Emotions | 134.139 | 66 | 0.919 | 0.904 | 0.084 [0.063, 0.104] | 0.066 |
| Life Satisfaction | 61.227 | 44 | 0.979 | 0.973 | 0.052 [0.009, 0.081] | 0.055 |
| Mindful Attention | 89.487 | 66 | 0.933 | 0.917 | 0.084 [0.059, 0.109] | 0.064 |
| Autonomy | 31.912 | 12 | 0.941 | 0.896 | 0.106 [0.062, 0.152] | 0.060 |
| Competence | 17.315 | 12 | 0.984 | 0.973 | 0.055 [0.000, 0.108] | 0.039 |
| Connectedness | 14.292 | 12 | 0.996 | 0.993 | 0.036 [0.000, 0.095] | 0.037 |
| Depression | 109.664 | 66 | 0.959 | 0.952 | 0.067 [0.044, 0.089] | 0.057 |
| Loneliness | 226.074 | 66 | 0.810 | 0.776 | 0.128 [0.110, 0.147] | 0.130 |
| Self-Esteem | 101.318 | 66 | 0.968 | 0.962 | 0.060 [0.035, 0.083] | 0.054 |
| Stress | 25.230 | 26 | 1.000 | 1.003 | 0.000 [0.000, 0.063] | 0.046 |
| Health | 109.762 | 44 | 0.886 | 0.858 | 0.101 [0.077, 0.125] | 0.083 |
| **Hypothesis 2. Social Diet vs. Both Controls:** | | | | | | |
| Positive Emotions | 223.392 | 66 | 0.939 | 0.928 | 0.095 [0.082, 0.109] | 0.055 |
| Negative Emotions | 163.534 | 66 | 0.932 | 0.920 | 0.075 [0.061, 0.090] | 0.053 |
| Life Satisfaction | 58.973 | 44 | 0.991 | 0.989 | 0.036 [0.000, 0.058] | 0.035 |
| Mindful Attention | 83.873 | 66 | 0.966 | 0.958 | 0.059 [0.039, 0.078] | 0.041 |
| Autonomy | 19.516 | 12 | 0.988 | 0.979 | 0.049 [0.000, 0.087] | 0.038 |
| Competence | 18.319 | 12 | 0.990 | 0.982 | 0.045 [0.000, 0.084] | 0.031 |
| Connectedness | 22.058 | 12 | 0.991 | 0.984 | 0.057 [0.014, 0.093] | 0.028 |
| Depression | 169.019 | 66 | 0.951 | 0.942 | 0.077 [0.063, 0.092] | 0.048 |
| Loneliness | 367.386 | 66 | 0.824 | 0.792 | 0.132 [0.119, 0.145] | 0.139 |
| Self-Esteem | 122.605 | 66 | 0.971 | 0.966 | 0.057 [0.041, 0.073] | 0.040 |
| Stress | 24.577 | 26 | 1.000 | 1.003 | 0.000 [0.000, 0.046] | 0.038 |
| Health | 166.086 | 44 | 0.873 | 0.841 | 0.103 [0.087, 0.120] | 0.080 |

Table S5 (Continued)

| Construct | χ^2^ | df | CFI | TLI | RMSEA [90% CI] | SRMR |
| --- | --- | --- | --- | --- | --- | --- |
| **Exploratory. Digital Diet vs. Social Diet:** | | | | | | |
| Positive Emotions | 168.056 | 66 | 0.906 | 0.889 | 0.104 [0.085, 0.124] | 0.067 |
| Negative Emotions | 146.272 | 66 | 0.880 | 0.858 | 0.092 [0.072, 0.112] | 0.071 |
| Life Satisfaction | 61.125 | 44 | 0.981 | 0.976 | 0.052 [0.009, 0.082] | 0.048 |
| Mindful Attention | 73.534 | 66 | 0.944 | 0.930 | 0.069 [0.039, 0.095] | 0.057 |
| Autonomy | 19.033 | 12 | 0.973 | 0.953 | 0.064 [0.000, 0.116] | 0.056 |
| Competence | 27.372 | 12 | 0.948 | 0.909 | 0.095 [0.047, 0.142] | 0.052 |
| Connectedness | 24.685 | 12 | 0.972 | 0.951 | 0.086 [0.036, 0.134] | 0.058 |
| Depression | 126.608 | 66 | 0.944 | 0.934 | 0.080 [0.059, 0.101] | 0.061 |
| Loneliness | 216.652 | 66 | 0.803 | 0.768 | 0.126 [0.108, 0.145] | 0.118 |
| Self-Esteem | 119.930 | 66 | 0.943 | 0.932 | 0.076 [0.054, 0.097] | 0.057 |
| Stress | 50.497 | 26 | 0.932 | 0.906 | 0.081 [0.047, 0.114] | 0.057 |
| Health | 86.280 | 44 | 0.911 | 0.889 | 0.082 [0.056, 0.107] | 0.075 |

*Note.* CFI = Comparative Fit Index. TLI = Tucker-Lewis Index. RMSEA = Root Mean Square Error of Approximation. SRMR = Standardized Root Mean Square Residual.

**Table S6**

*Second-Order Latent Growth Model Results*

| Variable | *b* | *b SE* | *p* |
| --- | --- | --- | --- |
| **Hypothesis 1. Digital Diet vs. Water Diet:** | | | |
| Positive Emotions | 0.06 | 0.13 | 0.655 |
| Negative Emotions | -0.13 | 0.09 | 0.145 |
| Life Satisfaction | 0.32 | 0.11 | 0.002 |
| Mindful Attention | 0.44 | 0.12 | <.001 |
| Autonomy | 0.24 | 0.09 | 0.007 |
| Competence | 0.25 | 0.10 | 0.014 |
| Connectedness | 0.08 | 0.11 | 0.482 |
| Depression | -0.08 | 0.08 | 0.363 |
| Loneliness | -0.17 | 0.07 | 0.016 |
| Self-Esteem | 0.30 | 0.07 | <.001 |
| Stress | -0.23 | 0.09 | 0.007 |
| Health | 3.16 | 1.99 | 0.112 |
| **Hypothesis 1. Digital Diet vs. No Diet:** | | | |
| Positive Emotions | -0.16 | 0.12 | 0.173 |
| Negative Emotions | 0.01 | 0.13 | 0.951 |
| Life Satisfaction | 0.33 | 0.13 | 0.009 |
| Mindful Attention | 0.33 | 0.13 | 0.008 |
| Autonomy | 0.16 | 0.09 | 0.070 |
| Competence | 0.24 | 0.11 | 0.023 |
| Connectedness | -0.04 | 0.11 | 0.733 |
| Depression | -0.10 | 0.09 | 0.274 |
| Loneliness | -0.13 | 0.11 | 0.246 |
| Self-Esteem | 0.22 | 0.08 | 0.005 |
| Stress | -0.15 | 0.09 | 0.094 |
| Health | 1.48 | 1.70 | 0.384 |
| **Hypothesis 1. Digital Diet vs. Both Controls:** | | | |
| Positive Emotions | -0.04 | 0.11 | 0.710 |
| Negative Emotions | -0.08 | 0.09 | 0.375 |
| Life Satisfaction | 0.32 | 0.10 | 0.002 |
| Mindful Attention | 0.36 | 0.10 | <.001 |
| Autonomy | 0.22 | 0.08 | 0.007 |
| Competence | 0.24 | 0.09 | 0.009 |
| Connectedness | 0.03 | 0.10 | 0.736 |
| Depression | -0.09 | 0.08 | 0.273 |
| Loneliness | -0.14 | 0.06 | 0.020 |
| Self-Esteem | 0.27 | 0.07 | <.001 |
| Stress | -0.17 | 0.07 | 0.024 |
| Health | 2.34 | 1.69 | 0.167 |

Table S6 (continued)

| Variable | *b* | *b SE* | *p* |
| --- | --- | --- | --- |
| **Hypothesis 2. Social Diet vs. Water Diet:** | | | |
| Positive Emotions | -0.05 | 0.14 | 0.711 |
| Negative Emotions | 0.10 | 0.10 | 0.302 |
| Life Satisfaction | 0.21 | 0.12 | 0.073 |
| Mindful Attention | 0.30 | 0.11 | 0.006 |
| Autonomy | -0.03 | 0.09 | 0.745 |
| Competence | 0.07 | 0.11 | 0.664 |
| Connectedness | -0.02 | 0.11 | 0.887 |
| Depression | 0.02 | 0.08 | 0.845 |
| Loneliness | -0.11 | 0.07 | 0.150 |
| Self-Esteem | 0.07 | 0.08 | 0.400 |
| Stress | -0.07 | 0.09 | 0.431 |
| Health | -0.22 | 1.94 | 0.909 |
| **Hypothesis 2. Social Diet vs. No Diet:** | | | |
| Positive Emotions | -0.27 | 0.13 | 0.043 |
| Negative Emotions | 0.34 | 0.14 | 0.014 |
| Life Satisfaction | 0.19 | 0.14 | 0.179 |
| Mindful Attention | 0.15 | 0.10 | 0.159 |
| Autonomy | -0.06 | 0.09 | 0.515 |
| Competence | 0.07 | 0.12 | 0.576 |
| Connectedness | -0.10 | 0.11 | 0.368 |
| Depression | 0.03 | 0.08 | 0.767 |
| Loneliness | -0.03 | 0.08 | 0.724 |
| Self-Esteem | -0.02 | 0.09 | 0.856 |
| Stress | -0.01 | 0.08 | 0.933 |
| Health | -1.98 | 1.83 | 0.279 |
| **Hypothesis 2. Social Diet vs. Both Controls:** | | | |
| Positive Emotions | -0.15 | 0.12 | 0.228 |
| Negative Emotions | 0.18 | 0.10 | 0.067 |
| Life Satisfaction | 0.20 | 0.11 | 0.074 |
| Mindful Attention | 0.23 | 0.10 | 0.018 |
| Autonomy | -0.04 | 0.08 | 0.662 |
| Competence | 0.06 | 0.10 | 0.518 |
| Connectedness | -0.04 | 0.10 | 0.691 |
| Depression | 0.02 | 0.08 | 0.832 |
| Loneliness | -0.08 | 0.06 | 0.193 |
| Self-Esteem | 0.04 | 0.08 | 0.594 |
| Stress | -0.04 | 0.07 | 0.595 |
| Health | -1.14 | 1.73 | 0.507 |

Table S6 (continued)

| Variable | *b* | *b SE* | *p* |
| --- | --- | --- | --- |
| **Exploratory. Digital Diet vs. Social Diet:** | | | |
| Positive Emotions | 0.10 | 0.15 | 0.475 |
| Negative Emotions | -0.25 | 0.13 | 0.047 |
| Life Satisfaction | 0.12 | 0.12 | 0.316 |
| Mindful Attention | 0.07 | 0.12 | 0.562 |
| Autonomy | 0.20 | 0.09 | 0.031 |
| Competence | 0.21 | 0.13 | 0.095 |
| Connectedness | 0.05 | 0.11 | 0.638 |
| Depression | -0.09 | 0.07 | 0.236 |
| Loneliness | -0.08 | 0.08 | 0.315 |
| Self-Esteem | 0.22 | 0.08 | 0.009 |
| Stress | -0.16 | 0.10 | 0.113 |
| Health | 3.39 | 1.77 | 0.055 |

*Note.* Positive *b*s suggest the treatment group (Digital Diet, Social Diet) reported greater increases than the reference group (Water Diet, No Diet, Both Controls, Social Diet). Negative *b*s suggest the treatment group reported greater decreases than the reference group. SOLGM analyses were not conducted for single-item variables (e.g., Objective Digital Media Time, Brief Happiness).
