# Supplementary material for: Does putting down your smartphone make you happier? the effects of restricting digital media on well-being
Source: PLoS One. 2024 Oct 14;19(10):e0306910. doi: 10.1371/journal.pone.0306910 (PMC11472914; doi:10.1371/journal.pone.0306910)
Supplement: Attachment — Submitted filename: Restricting Digital Media RESPONSE LETTER.docx [file pone.0306910.s002.docx]

Dr. Alastair Van Heerden

Academic Editor, *PLOS ONE*

Director: Community Based RC

Human Sciences Research Council

[avanheerden@hsrc.ac.za](mailto:avanheerden@hsrc.ac.za)

July 5, 2023

Dear Dr. Van Heerden:

The accompanying submission is a revised version of our manuscript PONE-D-23-01416R1, titled “Does Putting Down Your Smartphone Make You Happier? The Effects of Restricting Digital Media on Well-Being.” We are very grateful to you and the reviewers for the careful attention that our work received, and we were pleased to receive a revise-and-resubmit decision on the paper.

We are incredibly glad the reviewers thought our manuscript is clear and well-structured, as well as presents a study that is relevant, novel, and methodologically sound. We thank you and the review team for the positive responses and valuable feedback. The reviewers also pointed out some important ways that the manuscript could be improved. Overall, we believe the reviews allowed us to generate a clearer and better presentation of our findings.

Below, we detail how we have responded to the issues raised with the original submission, and in the revised manuscript you will see the specific changes we made to improve our work. For your convenience, we have resubmitted our manuscript in two versions: one clean version and one version with tracked changes (labeled accordingly). Throughout this letter, we have numbered and presented in italics the comments we received from the reviewers. Our responses to each comment follow in normal text.

**Response to Comment from Reviewer #1**

1. *Congratulations! The study is relevant, well-structured, methodologically quite sound, and provides novel and interesting findings presented in a clear and straightforward way and interpreted correctly. I particularly enjoyed reading the literature review - it is very informative and well-written. The discussion is relevant and to the point. Overall, the paper is so good that I do not have any suggestions for improvement: if it's not broken, don't fix it. Thank you for conducting this study and I'm looking forward to seeing it published.*

Thank you so much for your positive feedback and endorsement of our study. We greatly appreciate your recognition of the work that went into our literature review, as well as our methodology, findings, and discussion. We are also eagerly looking forward to seeing this study published so we can share our findings with the larger scientific community!

**Responses to Comments from Reviewer #2**

2. *Overall Structure: The manuscript is well-structured, starting with an introduction to the ubiquity of smartphones and digital media, followed by a review of existing literature on the impact of digital media and social media on well-being. The paper then presents the need for further research and the objective of the study.*

We appreciate your acknowledgement of the manuscript's overall structure. We aimed to create a logical flow from the broad context of smartphone and digital media use, to the specific research gaps in the literature, and finally to our study's objectives. Your positive feedback reassures us that we were relatively successful in this regard.

3. *Clarity: The manuscript is generally clear, but there is room for improvement in certain areas. The introduction section could benefit from a sharper focus on the relationship between smartphone use and well-being. Additionally, it may be helpful to provide more context on the specific research questions and hypotheses in the introductory section.*

We agree with your suggestions about further refining the clarity, focus, and context of the introduction of our manuscript. Accordingly, we have added a few additional paragraphs on pages 4-5 to the introductory section to address the issues raised above. We think these revisions will help provide readers a clearer understanding of the study's direction from the onset.

4. *Methodology: The methodology is well-described, with information on participant selection, study design, and measures used. The study employs a between-subjects design with four conditions: Digital Diet, Social Diet, Water Diet (active control), and No Diet (measurement-only control). The study focuses on Gen Z individuals and uses a range of well-established measures to assess well-being and related constructs.*

Thank you for acknowledging the clarity and robustness of our study's methodology, including our use of a between-subjects design and well-established measures.

5. *Results: The authors should provide more information on the randomization process, including how participants were allocated to different groups and any stratification factors used to ensure balance across groups.*

We have added another sentence to further explain the randomization process using the Qualtrics randomizer block (see page 16). Participants were assigned to condition using a Qualtrics randomizer block in the survey flow, with the "Evenly Present Elements" option deselected to maintain genuinely random assignment. In other words, there were no stratification factors used to balance across groups. This method, however, led to the creation of groups that were somewhat unequal in size.

6. *The sample size should be justified based on a power analysis or other relevant calculations, to ensure that the study has sufficient power to detect meaningful differences between groups. The authors should consider conducting a sensitivity analysis to assess the robustness of their findings to potential biases, such as attrition or nonresponse.*

We aimed to recruit 100 participants per condition based on a previous power analysis conducted by Vazire (2014) that gave general recommendations to the field. However, you make a smart suggestion to conduct a sensitivity power analysis, so we have done so and added it to page 15 of the manuscript.

7. *Other suggestions for improvement: The authors should address the potential for selection bias in their sample, given that the participants were recruited from a specific population (Gen Z individuals, mainly ages 18-25). They should discuss how this might limit the generalizability of their findings and consider conducting sensitivity analyses or subgroup analyses to account for potential biases.*

We appreciate your insight that our focus on Gen Z individuals ages 18-25 may limit the generalizability of our findings. Indeed, this is a significant consideration. Thus, we have included it as the first limitation in our manuscript on page 29, and expanded our discussion of the issue to address your concern.

8. *The authors could consider including a mediation or moderation analysis to investigate the potential mechanisms underlying the observed effects. This would help to provide deeper insights into how and why restricting digital media and social media use might influence psychological outcomes.*

Thank you for your suggestion about implementing mediation or moderation analyses to provide more in-depth insights into the mechanisms underlying the observed effects. We initially considered this and did undertake some exploratory moderator analyses. However, the multitude of outcomes, condition comparisons, and possible moderators led to an overwhelming number of analyses, without any discernible, consistent pattern. Consequently, these analyses appeared to introduce more confusion than clarity, which led us to exclude them from the manuscript. Regarding potential mediators, there are several theoretical mechanisms that have been proposed in previous research to explain the relationship between smartphone use and mental health, such as the Goldilocks hypothesis and the displacement hypothesis. However, the present study did not include any variables that would allow for an effective testing of these mechanisms. We appreciate your understanding and hope that the manuscript, in its current form, provides a useful contribution to the field. If the editor feels strongly that we should examine mediators or moderators, please let us know, and we could try to reconsider how we might address such analyses.

9. *In the discussion, the authors should consider the potential implications of their findings for policymakers, educators, and mental health professionals. For example, they could discuss how their results might inform strategies for promoting healthy digital media and social media use among young people and address the potential for digital interventions to improve psychological well-being.*

Excellent suggestion. We have added a paragraph to address potential implications for policymakers, educators, and mental health professionals (see pages 31-32).

10. *In the sentence "For ease of interpretation, difference scores are presented in raw (not log-transformed) form.", the term "raw" should be replaced with "untransformed" for clarity and precision.*

Thank you for the suggestion. We have revised the sentence accordingly.

11. *In the sentence "However, as recent revelations from The Wall Street Journal’s (2021) “Facebook Files” demonstrate, social media platforms are not always optimized for user well-being [61].", consider adding an explanation or brief summary of the "Facebook Files" for readers who may be unfamiliar with the issue.*

Good point. We have added some clarifying text to better describe the “Facebook Files” for readers unfamiliar with them.

12. *In the sentence "Of course, even if those small effect sizes are made detectable, the debate about whether such effects matter will likely continue.", consider rephrasing to "Of course, even if those small effect sizes are detected, the debate about whether such effects are practically significant will likely continue."*

We revised the sentence accordingly.

13. *In the sentence "Smartphone apps may merely represent tools that can be tailored for better or worse.", consider rephrasing as "Smartphone apps may simply represent tools that can be tailored for better or worse outcomes."*

We revised the sentence accordingly.

To conclude, we hope you agree that, after responding to these exceptionally thoughtful and constructive reviews, our revised manuscript offers a clearer, more transparent, and more valuable contribution to the literature. Needless to say, we would be happy to revise further in any ways that you think would improve the paper further. In the meantime, we eagerly await your editorial decision.

If you have any questions, please feel free to contact us directly.

Sincerely,

The Authors
