# Supplementary material for: Does putting down your smartphone make you happier? the effects of restricting digital media on well-being
Source: PLoS One. 2024 Oct 14;19(10):e0306910. doi: 10.1371/journal.pone.0306910 (PMC11472914; doi:10.1371/journal.pone.0306910)
Supplement: Attachment — Submitted filename: RDM R2 Response to Reviewers.docx [file pone.0306910.s003.docx]

Ali B. Mahmoud, Ph.D.
Academic Editor

PLOS ONE

May 15, 2024

Dear Dr. Mahmoud:

The accompanying submission is a revised version of our manuscript PONE-D-23-01416R2, titled “Does Putting Down Your Smartphone Make You Happier? The Effects of Restricting Digital Media on Well-Being.” We are grateful to you and Reviewer #3 for the additional feedback and glad to receive another revise-and-resubmit decision on the paper.

We are pleased that Reviewer #3 thinks the rigor of our manuscript is “of a very well-founded level.” Reviewer #3 also pointed out a few ways the manuscript could be further refined. We have carefully considered their suggestions and revised our manuscript accordingly. Overall, we believe this additional round of reviews further enhanced the presentation of our findings.

Below, we detail how we have responded to the issues raised with the original submission, and in the revised manuscript you will see the specific changes we made to improve our work. As directed, we have submitted:

- This rebuttal letter labeled “Response to Reviewers”
- A marked-up copy of our manuscript labeled “Revised Manuscript with Track Changes”
- An unmarked version of our revised paper without tracked changes labeled “Manuscript”

Throughout this response letter, we present in italics the comments we received from the reviewer. Our responses to each comment follow in normal text.

**Response to Comments from Reviewer #3**

*This document addresses a relevant topic about the study of happiness and the value that the use of digital media, social media, has on it. To do this, a comparison is made with the restrictive use of these two concepts and the use applied in some water applications in contrast to not having any restrictions. This in order to observe the differences and appreciate what effects they have on the perception of a state of well-being.

The authors make a methodological development very appropriate to the type of study they propose and apply strategies that are made up of statistical methods appropriate for the type of study.

They present results in accordance with the initial approaches and an exploratory approach is manifested for the type of study that aims to find the effects of the study in its analysis and in the relationships found between the constructs that make up each of the variables.

In its results, once the statistical studies are presented, a methodological and statistical rigor is evident that is very appropriate for the type of scientific product that is proposed for this journal. I consider that the paper meets an adequate quality to be considered for publication.*

We sincerely appreciate your thoughtful and detailed feedback on our manuscript. Your positive remarks regarding the methodological approach and statistical rigor of our study are immensely encouraging. Thank you for considering our paper suitable for publication.

*I recommend the following adjustments in its approach and development:
1. Establish a table of authors at the end of each of the sections where the study variables are defined. In this case, at the end of digital media, social media, water restrictions, and where appropriate, support for the scenario where there is no type of restriction. In this table, I recommend including the most relevant previous documents that support the theoretical model on which the proposed model is based.*

Thank you for your insightful suggestion to include a table of authors at the end of each section where the study variables are defined. While we initially thought that adding such tables directly into the Introduction might disrupt the flow of the text for readers, we recognized the value of your recommendation. Therefore, we have added a comprehensive table to the Supplemental Materials (see Table S1) that includes correlational and experimental studies relevant to digital media and social media, along with their relevant outcomes and key findings. This table effectively highlights the pertinent outcomes each previous study has examined and clarifies our rationale for selecting the variables in the present study.

Table S1 specifically addresses digital media and social media, as the water restriction component was only included as an active control. We have also revised the Supplemental Materials Table numbers (S1-S6) accordingly and noted the addition of the new table (S1) at the end of the Introduction on pages 11-12. We believe this approach maintains the narrative flow while providing a valuable resource for readers. Thank you again for this excellent suggestion.

*2. Add a graphic diagram to illustrate the development of the methodological diagram developed during this document. To do this, add in each phase an explanation about the inputs from the previous phase and what elements or values are delivered in the next step, as a process diagram.*

We appreciate your astute suggestion to include a graphic diagram detailing our methodology. Per your recommendation, we have added a CONSORT process flow diagram that visually illustrates each phase of our experiment. This diagram, now featured as Figure 1 on page 15, clearly outlines the inputs and outputs at each stage, providing a cohesive overview of our experimental design. We believe this addition greatly enhances the clarity and accessibility of our methodological approach for readers.

*3. In your conclusions, delve into the effects that develop from the findings in your study, what elements are added to the literature, and what gaps are closed with the results of this research, likewise, in case they are projected new forms of questions, new methods or elements that propose improvements in the work proposals, include them in your explanation of future work and confirmation of obtained results.*

Thank you for this comment regarding the need to delve deeper into the effects of our study’s findings and their contributions to the literature. While we have dedicated substantial discussion to new methods and proposals for future improvements in the Limitations and Future Directions section on pages 34-36, we recognize the importance of explicitly highlighting the unique contributions and gaps addressed by our research. We did this to some extent in the Introduction but recognize that it should be reiterated in the Discussion section.

To address your comment, we have added a new paragraph to the Discussion section on pages 32-33. This paragraph details how our study advances the field by (1) employing an experimental design with general restrictions over an extended period; (2) utilizing objective measures of smartphone use; (3) assessing a broader range of social media apps; (4) comparing the effects of smartphone vs. social media restriction; (5) examining outcomes not previously assessed in other restriction studies; and (6) implementing a stronger neutral activity control group to better control for demand effects. These additions aim to underscore the significant contributions of our study and the gaps it helps to close in the existing literature.

*4. Expand the explanation of the lines of research that open from this research, as well as the elements that are required for these results to be extrapolated or generalized in public policies or in new conditions that promote well-being*

In response to your comment regarding the expansion of the explanation of the lines of research opened by this study and the elements required for the results to be extrapolated or generalized in public policies or new conditions that promote well-being, we have expanded the relevant paragraph in the discussion section to provide a more comprehensive overview of how our findings can inform future research, policy-making, and practical applications. Specifically, we elaborated on potential policy measures, such as setting screen time limits for minors and encouraging balanced digital practices in schools and workplaces. We also outlined how educators could incorporate digital literacy programs into their curricula and suggested specific therapeutic techniques for mental health professionals based on our findings. Finally, we identified key future research directions, such as longitudinal and cross-cultural studies, to further investigate the long-term and diverse impacts of digital media use on well-being. We believe these additions provide a clearer and more detailed response to your comment, demonstrating the broader applicability and potential impact of our research findings. Please see revisions on pages 36-37.

*I consider that these are minor changes, in the theoretical, methodological, and technical aspects, it seems to me that the rigor is of a very well-founded level.

Analyzing the impacts of restrictions on digital media, social media, water usage, and a scenario with no restrictions can indeed contribute to our overall happiness. Restrictions on digital media and social media can curb information overload, enhancing mental well-being. Water usage restrictions foster sustainability which supports a healthier environment and future generations' happiness. However, a complete absence of restrictions may lead to detrimental consequences in both digital and environmental domains, affecting our overall contentment. Hence, a balanced approach that combines responsible usage with thoughtful regulation can promote happiness by safeguarding our well-being and the planet's health.*

Thank you again for your constructive and useful feedback, Reviewer #3. Your suggestions for enhancing the theoretical, methodological, and technical aspects of the manuscript were well-received and greatly appreciated.

To conclude, we hope our revisions, guided by Reviewer #3’s exceptionally thoughtful feedback, have enhanced the manuscript sufficiently to satisfy the publication criteria of PLOS ONE. Of course, we are open to any further revisions you require. In the meantime, we eagerly await your editorial decision.

If you have any questions, please feel free to contact us directly.

Sincerely,

The Authors
